# Multiple evoked and induced alpha modulations in a visual attention task: Latency, amplitude and topographical profiles

**DOI:** 10.1371/journal.pone.0223055

**Published:** 2019-09-26

**Authors:** Manuel Vázquez-Marrufo, Macarena García-Valdecasas, Rocío Caballero-Diaz, Ruben Martin-Clemente, Alejandro Galvao-Carmona

**Affiliations:** 1 Experimental Psychology Department, Faculty of Psychology, University of Seville, Seville, Spain; 2 Signal Processing and Communications Department, Higher Technical School of Engineering, University of Seville, Seville, Spain; 3 Department of Psychology, Loyola Andalucía University, Seville, Spain; Universita degli Studi di Roma La Sapienza, ITALY

## Abstract

Alpha event-related desynchronization (ERD) has been widely applied to understand the psychophysiological role of this band in cognition. In particular, a considerable number of publications have described spectral alterations in several pathologies using this time-frequency approach. However, ERD is not capable of specifically showing nonphase (induced) activity related to the presentation of stimuli. Recent studies have described an evoked and induced activity in the early phases (first 200 ms) of stimulus processing. However, scarce studies have analyzed induced and evoked modulations in longer latencies (>200 ms) and their potential roles in cognitive processing. The main goal of the present study was to analyze diverse evoked and induced modulations in response to visual stimuli. Thus, 58-channel electroencephalogram (EEG) was recorded in 21 healthy subjects during the performance of a visual attention task, and analyses were performed for both target and standard stimuli. The initial result showed that phase-locked and nonphase locked activities coexist in the early processing of target and standard stimuli as has been reported by previous studies. However, more modulations were evident in longer latencies in both evoked and induced activities. Correlation analyses suggest that similar maps were present for evoked and induced activities at different timepoints. In the discussion section, diverse proposals will be stated to define the potential roles of these modulations in the information processing for this cognitive task. As a general conclusion, induced activity enables the observation of cognitive mechanisms that are not visible by ERD or ERP modulations.

## Introduction

Diverse domains have been studied in the electroencephalographic response during cognitive tasks, including a time domain (event-related potentials (ERPs)) and a frequency domain, among other domains [1]. In the second domain, multiple technical approaches have been used to analyze spectral modulations during cognitive processing. In particular, time-frequency techniques have shown the evolution in the millisecond range of spectral bands (e.g., alpha, beta, and theta) in multiple cognitive paradigms [2, 3, 4]. One of these techniques was defined as event-related desynchronization (ERD) [5, 6]. When it was first developed, alpha ERD was interpreted as a decrement of the synchrony of the alpha band (assuming alpha oscillation as an idling status for neural networks); therefore, a reduction would mean an activation of the neural tissue. Another option described at the beginning of this technical approach was the event-related synchronization (ERS). This concept was intended to represent, in the case of the alpha band, a higher synchronized activity for this frequency and consequently a relaxation of the neural structures involved [7].

From then to the present, multiple studies have applied these interpretations to understand diverse modulations that occurred during diverse cognitive tasks [8, 9, 10]. ERD is calculated by filtering the EEG signal in the desired band, squaring (in ERD) or rectifying (in a similar technique defined as Temporal Spectral Evolution (TSE)) the signal (to prevent cancellation in the averaging process) and ultimately averaging desired EEG trials [5, 11, 12]. Because of this procedure, ERD or TSE mixes phase (evoked) and nonphase (induced) activities in its trace. At this point, it is necessary to clarify that diverse definitions have been used for the term “induced activity” [13]. In some cases, the term is simply related to single trial power, whereas in other studies, phase-locked activity is additionally removed as it has been done in the present study.

Induced activity analysis has not been applied extensively in the study of cognition as ERD [14, 15, 16]. The application of this analysis could help to better define the modulations that appear in the interval that follows the onset of the stimulus. For instance, in a study by our group [17], an alpha-TSE modulation occurred from 300 to 500 ms after the onset of a stimulus. This modulation was higher in the attended stimuli than in the unattended stimuli and was interpreted as a postperceptual effect for attended stimuli. Several studies have reported similar results [18, 19].

Calculating the phase and non-phase activities separately could help to determine whether the modulation of the induced activity occurs even prior to the postperceptual period and could better define the timing of cognitive mechanisms involved in information processing. With regard to the comparison between ERD and TSE, the former is calculated using “squaring” and the latter with a rectification function. Hari et al [20] indicated that the TSE method maintains the same amplitude after transformation (not powering the wave) and consequently guarantees a direct comparison between phase-locked and non–phase-locked activity.

Previous studies have described phase and non-phase modulations of the alpha band in different cognitive tasks (e.g., attention, motor, and memory) [11]. Moreover, early evoked and induced activities have been highlighted in the middle of a debate regarding different hypotheses for alpha psychophysiological meaning. Sabate et al [21] describe three potential hypotheses for alpha in stimuli processing: alpha is an active facilitation of cortical processing of the stimuli (processing hypothesis), some authors suggest an inhibitory role for this band, and other authors propose a reduction of the alpha wave to increase the signal-to-noise ratio (inhibition-timing hypothesis). In a comprehensive review of this topic, Klimesch et al [11] indicated that alpha modulations (evoked and induced) were present during the 200 ms after the onset of the stimuli. However, modulations that occur even later than 200 ms have not been extensively analyzed in the evoked and induced waves [22]. Therefore, the main aim of the present study was to analyze diverse induced and evoked modulations (based on different parameters, such as latency, amplitude (sign) and topography) in the processing of target and standard stimuli in a visual oddball task even later than the first 200 ms. Once they are parameterized, a second aim will be to investigate whether some of these components are closely related (topographically), suggesting a potential relationship between phase and non-phase activities or even inside evoked or induced modulations.

### Predictions

In the current study, an ample collection of induced and evoked modulations is expected in the information processing of target or standard stimuli during the execution of an oddball task. All of the induced and evoked modulations will show diverse latencies, amplitudes (sign) and topographical profiles. In the particular case of induced modulations, these modulations will exhibit different latencies and topographies compared to the ERD modulations. A precise observation of the non-phase activity will proportionate a better knowledge of the timing of cognitive mechanisms in information processing. Another prediction is that some evoked and induced modulations could be highly correlated (in latency and topography) and could suggest a certain functional relationship for these activities. Moreover, it could also be possible to identify topographical correlations internally in the evoked or induced domains, suggesting that some cognitive mechanisms are reactivated during information processing.

## Materials and methods

### Participants

The current study was carried out in compliance with the Declaration of Helsinki. All participants were informed of all relevant information related to their participation of the study and had at least seven days to decide whether they wanted to be participants and sign informed consent. The experimental protocol was approved by the ethics committee of the University of Seville (project code: PSI2016-78133-P). Twenty-one adults were recruited from university students, faculty and staff (age: 21 to 45 years; mean: 28.4 ± 7.94) (gender: 12 women and 9 men) (handedness: all were right handed participants with the exception of two left handed participants). All subjects were in good health and without a significant neurological or drug consumption history.

### Cognitive task

The cognitive task used was a “visual oddball” in which the subject must discriminate uncommon visual stimuli (target) (probability: 25%) in a sequence of frequent stimuli (standard). The target stimulus consisted of a rectangle with a checkerboard pattern that comprised red and white squares. The standard (frequent) stimulus was equivalent in size with the same pattern but with black and white squares. All stimuli subtended a 7.98° × 9.42° visual angle at a viewing distance of 80 cm. Both stimuli were displayed randomly in the center of the screen. When a target was displayed, the subject was required to press the mouse button with the right index finger and ignore the standard stimulus. All stimuli were presented for 500 milliseconds (ms), and the stimulus onset asynchrony (SOA) was one second, during which the subject could respond. A fixation point was present during the SOA to avoid changes in eye position during the experiment. As suggested by Klimesch et al [14], one block with at least 200 trials was used to obtain a good performance in a target/standard task. At the end of the experimental session, the reaction time and percentage accuracy (for the target stimuli and overall, including no responses for the standard stimuli) were calculated. All participants were asked to respond as quickly and accurately as possible.

### EEG procedure

**Fig 1:**
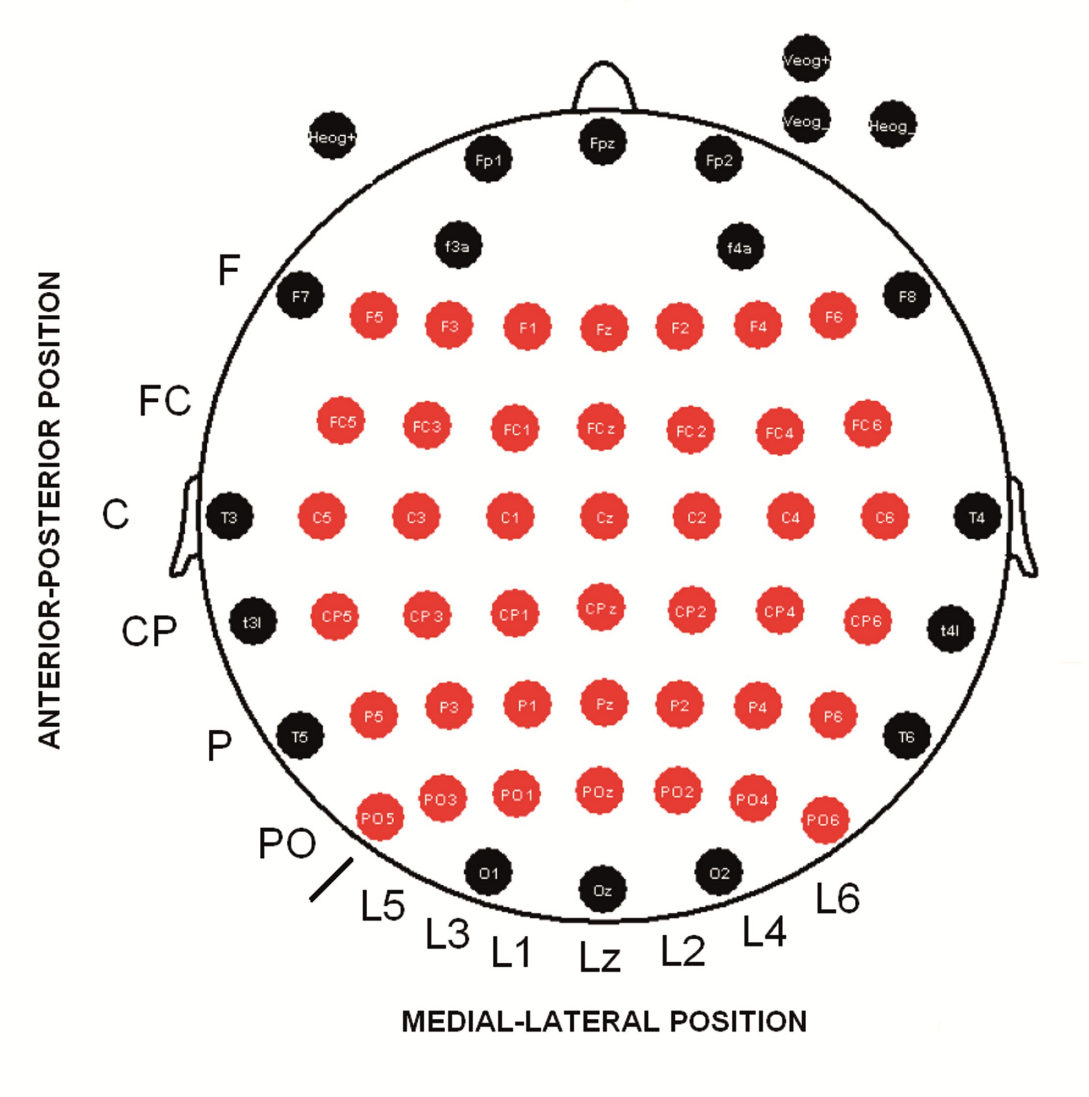
Position of 58 scalp electrodes for EEG recording. Red labels indicate derivations that were used for computing statistical analysis (ANOVA and correlation analyses for voltage maps).

The electroencephalogram was recorded from 58 scalp electrodes referenced to the linked earlobe channel and offline rereferenced to an averaged reference (see Fig 1 for detailed locations of recording derivations). To assess eye movements, 4 additional channels were added to the standard montage as follows: two electrodes were placed 1 cm from the outer canthi of each eye (HEOG), and two electrodes were placed above and below the right eye (VEOG). The EEG signal was amplified using BrainAmp amplifiers (Brain Products GmbH, Germany) and digitally stored using Brain Vision Recorder software (Brain Products GmbH, Germany). Recording parameters were established in a digitization rate of 500 Hz and filtered in a bandpass of 0.01–100 Hz with impedance values maintained below 5 kOhm during the experiment.

The EEG signal was processed with the following steps: ocular correction for blinking artifacts in the scalp electrodes using the algorithm developed by Gratton et al [23]; epoching of the continuous EEG recording (-200 to 1000 ms, zero being the onset of the target stimulus); baseline correction calculated from -100 to 0 ms; and visual inspection of EEG epochs and rejection of interval with artifacts. Moreover, the trials in which the HEOG signal was outside the ±75 μV range were rejected. After this processing, averaging was performed to achieve ERP waves. Next, a bandpass filter was subsequently applied in two alpha bands defined by the individual alpha frequency (IAF) procedure [14] (8–10.5 and 10.5–13 Hz) to obtain an evoked response (phase activity) for both the target and standard stimuli.

Furthermore, to calculate the temporal spectral evolution (TSE), an identical bandpass filtering in previously defined alpha bands (8–10.5 and 10.5–13 Hz) was performed over EEG epochs, followed by rectifying the signal and finally averaging for the stimulus condition (target and standard) [17]. A subtraction of evoked activity from TSE was subsequently performed to calculate the induced response (non-phase activity).

The first peak for evoked activity was identified as the maximum positive value in the interval between 100 to 200 ms (identified in the Oz electrode). The induced activity showed a first “valley” for which its latency was calculated for the maximum negativity in the interval from 100 to 200 ms (also identified in the Oz electrode). For better determination of the peaks or valleys, a low pass filter (5 Hz (48 dB/octave)) was used to eliminate small frequency fluctuations, as described in a previous study [17]. After the latency was determined, the amplitude values for the remaining electrodes were exported in the same latency for the topographical study, as some authors suggest [24]. To calculate the potential differences between the evoked and induced activities in absolute terms, the last one was transformed in positive values.

Because an interplay could occur between the evoked and induced activity, especially affecting the amplitude parameter, we have checked that the induced modulation was truly non–phase-locked activity. To do so, the evoked response was estimated through averaging over trials and then subtracted from each of the individual trials, or epochs, to exclude any evoked contribution when analyzing the induced activity [13, 25]. The trials were filtered in the desired bands (8–10.5 and 10.5–13 Hz) and Hilbert transform was applied to calculate instantaneous phase. The phases of induced activity were measured at 120 ms in single trials of target stimulus presentation, corresponding to the estimated grand average latency of the induced response. In addition, for the purposes of comparison, we also calculate the phases of the evoked responses (individual averages) by following an equivalent procedure (at the latency of maximum activity (135 ms)).

Aside from the first peak of evoked activity and the first valley for induced activity, other modulations in the grand averages were observed in longer latencies for evoked and induced activities and in target and standard stimuli (>300 ms). However, these modulations were not consistently present in all subjects, which meant that no latency analysis could be performed. To analyze the potential relationship between these modulations, voltage maps were calculated for both the evoked and induced activities. Map values were exported in the peak and valley latencies observed in the grand average. No interpolation procedures were applied to preclude modifications of the data that could affect further topographical analysis.

### Statistical analysis

To analyze the potential differences in the latency for the first evoked peak and induced valley, an analysis of variance (ANOVA) was calculated with the following factors: 1) TYPE of stimuli (levels (2): Target and Standard); 2) ACTIVITY (levels (2): Evoked and Induced).

For the study of topographical differences in the amplitude of the early evoked and induced activities and between the target and standard stimuli, an ANOVA was applied with the following factors: Factor 1: “TYPE of stimuli” (levels (2): target and standard); Factor 2: “ACTIVITY” (levels (2): evoked and induced); Factor 3: “Anterior-posterior Position” of the electrode (levels (6): Frontal; Frontocentral; Central; Centroparietal; Parietal; Parietooccipital); and Factor 4: “Lateral-Medial Position” (levels (7): from lateral left to lateral right, example: Line 5, Line 3, Line 1, Midline or Line zero (z), Line 2, Line 4, Line 6) (i.e., F5, F3, F1, Fz, F2, F4, and F6) (see Fig 1 for a description of factors and electrode positions analyzed). A Greenhouse-Geisser correction for sphericity was applied. A Bonferroni correction was carried out in multiple comparisons for the post hoc analysis. A probability of p < 0.05 was considered significant.

To analyze the correlations between voltage maps, a Pearson’s product-moment r was employed. Voltage maps were calculated in the peak and valley latencies observed in the grand averages for both the target and standard. The typical significance level (0.05) was divided by the number of comparisons made (20), as described by Kileny and Kripal [26] to obtain an adjusted significance level (p = 0.0025).

## Results

### Behavioral

In the analyses of the behavioral variables, the following results were obtained: reaction time: 318 ± 34.3 ms; accuracy for the target stimulus: 99.2 ± 1.8; and accuracy of global performance: 97.1 ± 4.79. The high values of accuracy in the target and global performance (responding to target and avoiding standard responses) indicates that the subjects adequately followed the experimenter instructions to be accurate during all experiments.

### Evoked-induced target-standard (latency)

**Fig 2:**
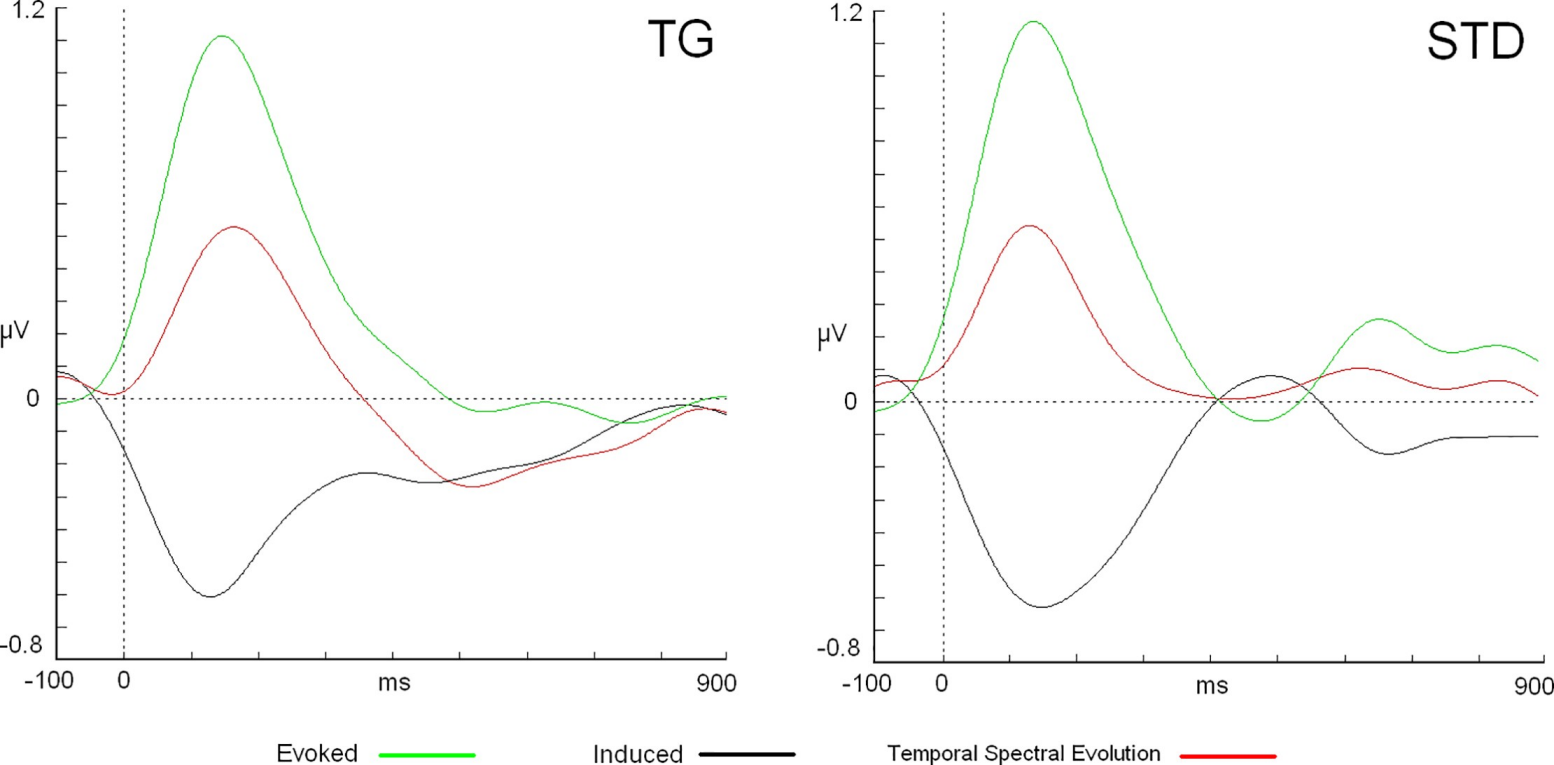
Comparison among evoked, induced and TSE waves for target and standard stimuli. Traces correspond to Oz and low alpha band (8–10.5 Hz).

In the low alpha (8–10.5 Hz), a double interaction between the “TYPE” of stimuli and “ACTIVITY” factors was significant (F(1,20 = 10.02, p = 0.004) (ƞ^2^ = 0.334) for the latency parameter. This effect was caused by a faster latency of the induced activity (120 ± 36.2 ms) in the target stimuli than that of the evoked activity (135 ± 40.3 ms) (p = 0.038), which did not occur in the case of the standard stimuli (induced: 148 ± 41.9 ms; evoked: 141 ± 26.9 ms) (see Fig 2). This result could also be interpreted as a difference in the latency in the induced activity between the target and standard, whereas no difference was found for the evoked activity between both types of stimuli.

Furthermore, in the high alpha (10.5–13 Hz), there was a trend for the interaction of the TYPE of stimuli and ACTIVITY factors for the latency (F(1,20) = 3.32, p = 0.083) (ƞ^2^ = 0.143). The induced activity of the target was again faster than that of the standard (induced), with limited difference between evoked for both types of stimuli (evoked-target: 125 ± 33 ms; evoked-standard: 132 ± 34.1 ms; induced-target: 127 ± 34.5 ms; induced-standard: 147 ± 45.1 ms).

### Evoked-induced target-standard (amplitude)

With regard to the amplitude parameter, ANOVA confirmed a statistically significant effect for the ACTIVITY factor for both alpha bands as follows: 1) low-alpha: (F(1,20) = 87.5, p<0.001) (ƞ^2^ = 0.814) and high alpha (F(1,20) = 57.1, p<0.001) (ƞ^2^ = 0.741). In both cases, the differences were explained by a higher amplitude of the evoked activity (low alpha: 1.57 ± 0.57 μV; high alpha: 1.26 ± 0.46 μV) than that of the induced activity (low alpha: -0.77 ± 0.40 μV; high alpha: -0.76 ± 0.38 μV) (see Fig 2). It is necessary to note that the induced activity was transformed in positive values to compare both activities in absolute terms for ANOVA.

### Topographic correlation analyses

**Fig 3:**
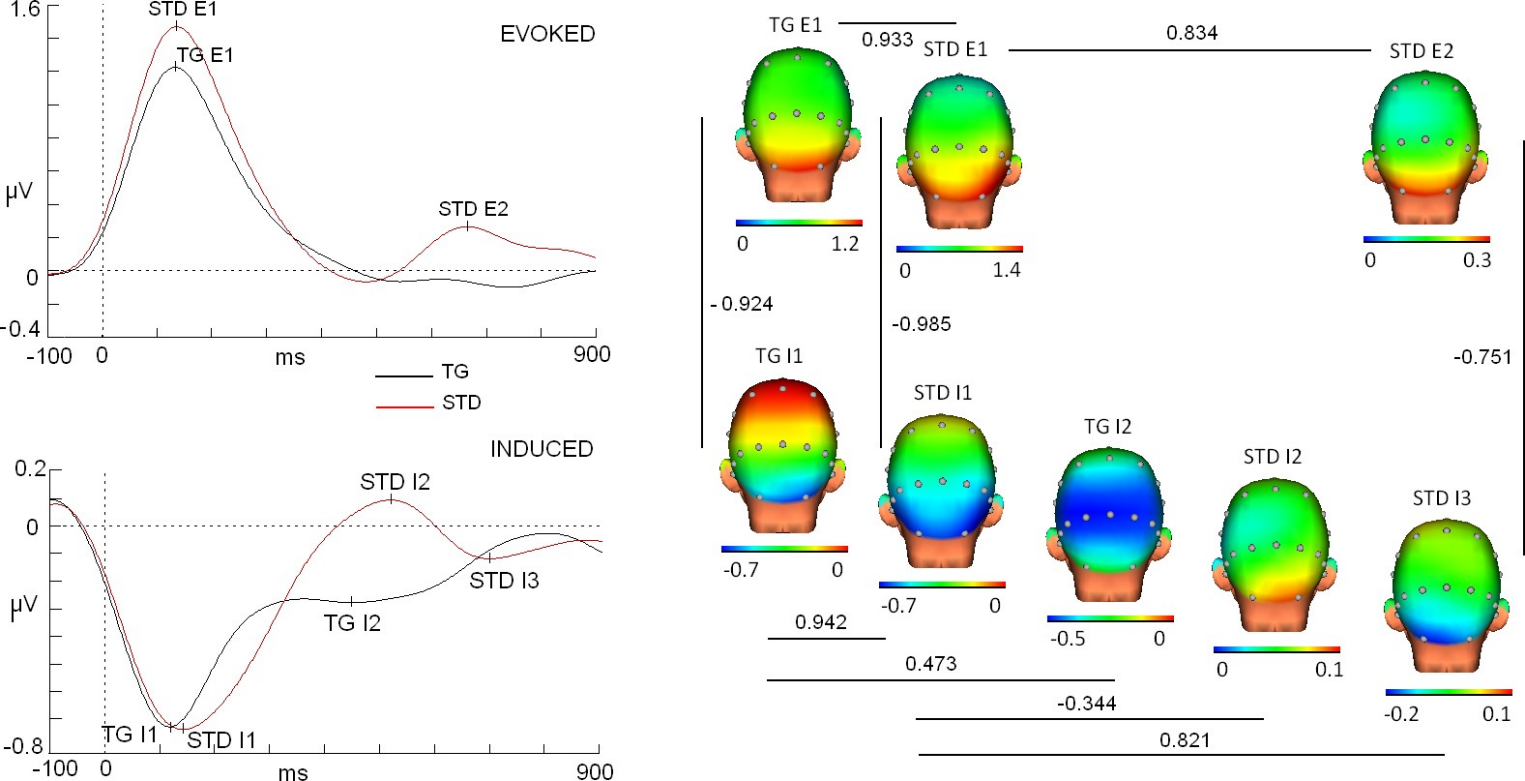
Maps and correlation scores of evoked and induced modulations in target and standard stimuli and for low alpha band (8–10.5 Hz). Note that scales were individually adjusted to evidence similar topographies but different amplitudes. Traces correspond to Oz derivation.

**Fig 4:**
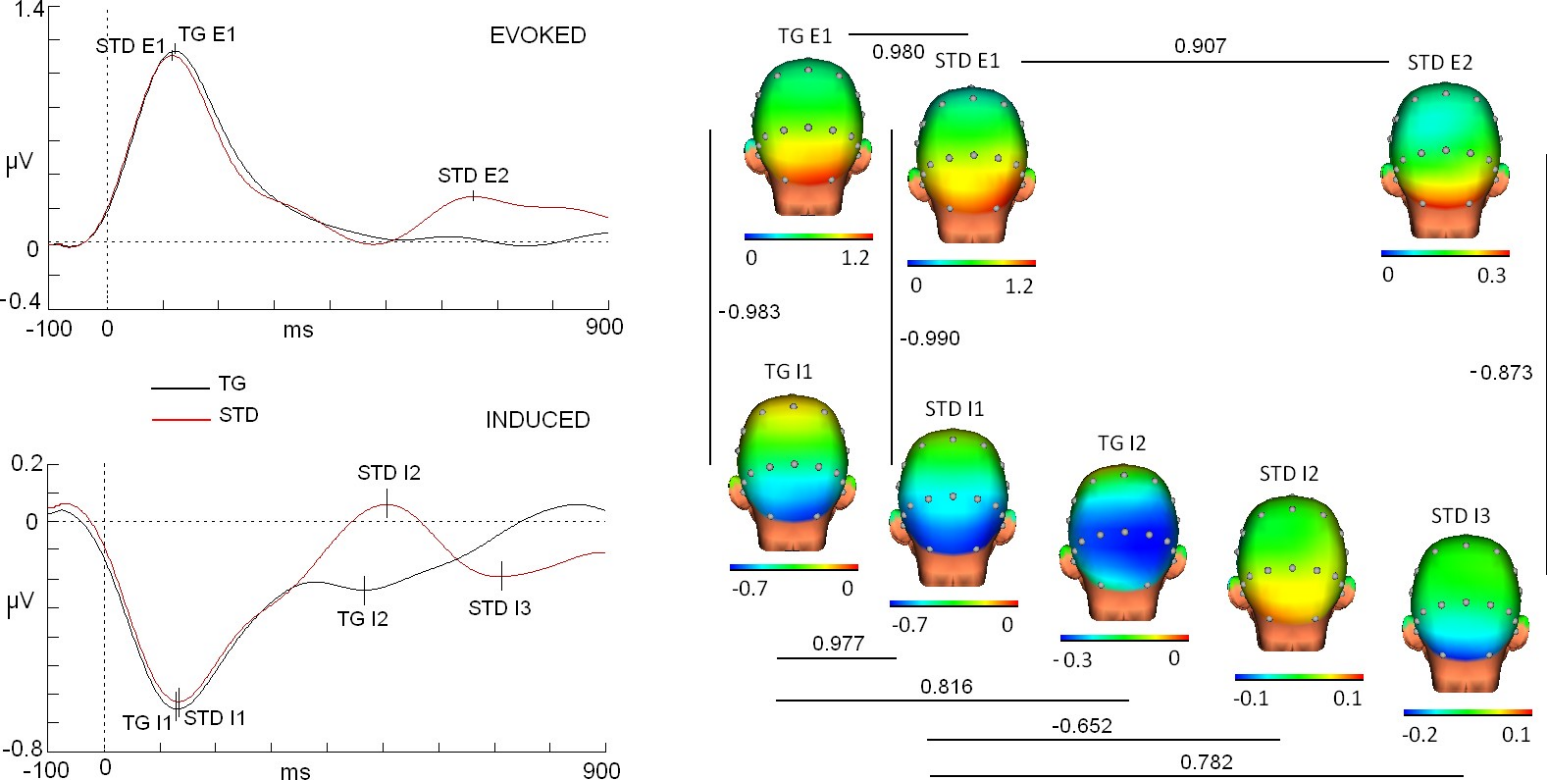
Maps and correlation scores of evoked and induced modulations in target and standard stimuli and for high alpha band (10.5–13 Hz). Note that the scales were individually adjusted to evidence similar topographies but different amplitudes. Traces correspond to Oz derivation.

Multiple correlation analysis indicated diverse results regarding the topographical profiles of evoked and induced modulations (see Figs 3 and 4 for maps and values).

First, the correlation scores were excellent (>0.9) for different comparisons between the evoked and induced first modulations (<200 ms) in both the target and standard, and for both alpha bands. For instance, the map of the first peak of evoked activity for the target in low alpha (latency: 132 ms) showed an excellent correlation score with the map of the first peak for the standard stimuli (latency: 134 ms) (r: 0.933, p<0.0025). In the case of high alpha, similar results were obtained between the map of the first peak for the target (latency: 120 ms) and the map of the first peak for the standard stimuli (116 ms) (r: 0.980, p<0.0025).

With regard to the induced activity in the low alpha band, the map for the first valley of the target (latency: 120 ms) correlated with an excellent score with the map of the first valley of the standard (latency: 144 ms) (r: 0.942, p<0.0025). A similar result was also obtained for the same comparison in the high alpha (latency first target valley: 124 ms; latency first standard valley: 132 ms) (r: 0.977, p<0.0025).

When the evoked and induced activities were correlated, excellent scores were also obtained but in this case with a negative sign. In the case of the low alpha band, correlation scores were identified including r: -0-924 (p<0.0025) for targets (latency first evoked peak: 132 ms; latency first induced valley: 120 ms) and r: -0.985, p<0.0025 for standards (latency first evoked peak: 134 ms; latency first induced valley: 144 ms). In the case of the high alpha band, even higher correlation scores were identified for the following comparisons: r: -0.983, p<0.0025 for targets (latency first evoked peak: 120 ms; latency first induced valley: 124 ms) and r:-0.990, p<0.0025 for standards (latency first evoked peak: 116 ms; latency first induced valley: 132 ms).

With regard to the correlation between the early and late evoked activity for standards, the low alpha band exhibited a good correlation score (r: 0.834, p<0.0025) (latency first evoked peak: 134 ms; latency second evoked peak: 660 ms). In the case of high alpha, these values were also at an excellent level (r: 0.907, p<0.0025) (latency first standard peak: 116 ms; latency second standard peak: 654 ms).

In the case of the comparisons made for the induced activity in different latencies, diverse correlation scores were identified. For the low alpha band, the comparison in targets between the first valley (120 ms) and a second valley (448 ms) resulted in a r: 0.473 (p<0.0025). An even lower correlation score (r: -0.344, p = 0.02, n.s.) was identified for standards when the first valley (144 ms) was compared with a second peak (518 ms). Finally, a good correlation score was identified in the standards between the first valley (144 ms) and a second valley (698 ms) (r: 0.821, p<0.0025). In high alpha, we identified differences compared to the low alpha values. In particular, the first and second valleys of targets (124 and 440 ms) exhibited higher correlation scores than those of the low alpha band (r: 0.816, p<0.0025). The comparison between the first valley (132 ms) and a first peak (522 ms) for standards also showed a higher correlation score (r: -0.652, p<0.0025). Finally, the first (132 ms) and second (728 ms) valleys for standards exhibited a lower correlation score than low alpha (r: 0.782, p<0.0225).

### Phase analysis for evoked and induced activity

**Fig 5:**
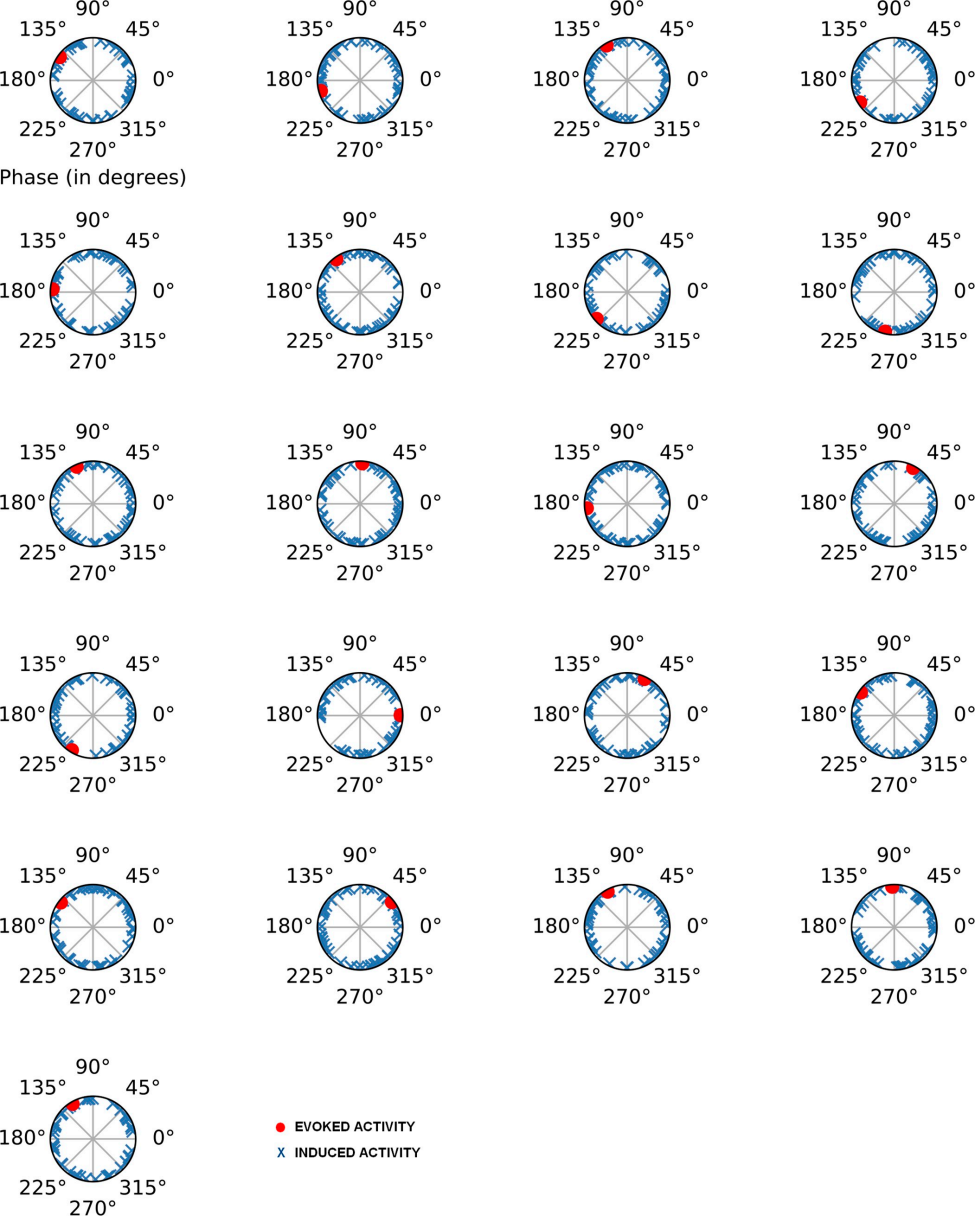
Polar plot of the phase analysis for the evoked and induced activity from target stimuli and for low alpha (8–10.5 Hz) in each subject. Red dots represent phase values of evoked activity, and blue crosses represent phase values of induced activity. Evoked activity calculations were made for each individual average, and induced activity was calculated for each subject in each valid single trial.

**Fig 6:**
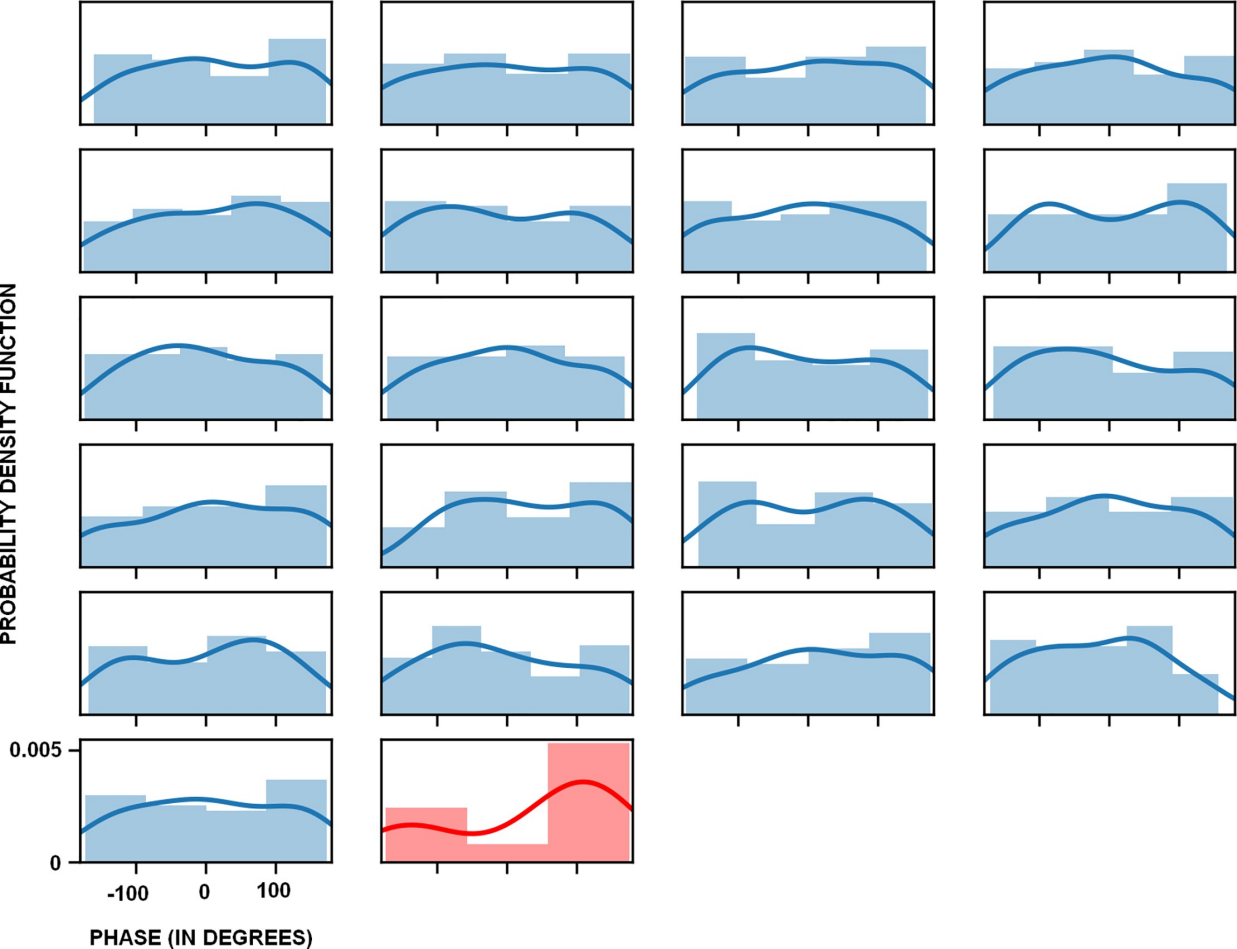
Histograms and probability density functions for phase values of evoked and induced activity. Note that the phase of the induced activity is randomly presented, in contrast to a more unimodal presentation for the evoked activity.

After performing phase analysis of induced and evoked activity, it is possible to check that a unimodal distribution of phase values is present in the evoked activity (see Figs 5 and 6). On the other hand, induced activity shows a random distribution in its phase values according to the single trial analysis. The difference between the unimodal phase range for the evoked activity and the random phase values for the induced activity suggest that it is possible to discard the potential contribution of the evoked activity over the induced activity to the amplitude values and vice versa. Therefore, the induced activity and its potential modulations are likely a truly non–phase-locked activity and are not caused by the jittering of the evoked activity.

## Discussion

Behavioral data (reaction time) showed characteristic values for this cognitive task as has been detailed in previous studies with similar experimental conditions [27, 28]. Subjects were accurate (approximately 99%) and responded with latencies of approximately 300 ms after the onset of the target stimuli.

In the analysis of the EEG frequency domain, the first evident result is that alpha TSE modulation showed negative values as is common in this type of cognitive task approximately 300 to 500 ms after the onset of the stimuli (not analyzed in this study) (see Fig 2). This modulation has been observed in previous studies with diverse cognitive tasks and TSE or ERD analysis [17, 18, 19]. However, when the evoked activity was subtracted from the TSE wave, the first valley in the nonphase activity was present well before the TSE modulation (in the first 200 ms after stimuli onset).

With valley latencies between 118 and 152 ms, a decrease of alpha non-phase activity occurs in a similar latency compared to the first peaks of the evoked response (120–138 ms), as described in previous studies [11, 21]. However, evoked and induced activity was not restricted to early phases of the stimulus response. In the present study, it was also possible to observe in the induced activity a second valley for target stimuli and a peak-valley complex for standard stimuli in longer latencies (> 300 ms). Therefore, it appears highly recommendable to calculate the induced activity once TSE has been computed to precisely observe non-phase activity related to cognitive processing that is hidden in the TSE trace.

### Evoked and induced early activities (<200 ms)

With regard to the latency comparison including the type of stimuli (target and standard) and the activity (evoked and induced), no differences were found for evoked modulations (between target and standard); however, a shorter latency was obtained for the induced valley of the target stimuli than the standard stimuli (statistically significant for low alpha and a trend for high alpha). One potential interpretation would be that the faster latency for the reduction of the alpha band (in non-phase activity), even before the evoked response reaches its peak, could serve as an efficient increment of the signal-to-noise ratio, as has been suggested by other authors [11, 21]. Therefore, it could be possible to argue that a preceding induced modulation (shorter latency) compared to the evoked response suggests a top-down process to improve stimulus processing to reduce “alpha noise”. This hypothesis is in accordance with other studies that have proposed that alpha suppression does not reflect a bottom-up process but is, in contrast, based on a top-down mechanism [11, 29]. Indeed, Klimesch et al [11] suggest that the latency close to the ERP component of P1 is when top-down and bottom-up processes interact and induced activity could “shape” the evoked modulation caused by the incoming stimuli.

A surprising result was related to the type of stimulus (target and standards) and non-phase activity. The decrement of induced alpha was sped up only for targets (and not for standards), which suggests a potential modulation based on the relevance of the stimuli for the task. This evidence supports that the determination of the relevance of a stimulus occurs in the early phases of information processing. In particular, induced activity in the target stimuli reaches its peak at 120 ms compared to 144 ms for the standard. Therefore, this induced activity could be preceded and potentially linked to earlier processes of categorization of the relevance of the stimuli. Several studies have suggested that a gating system for relevance is engaged in the first phases of information (approximately 50–70 ms) in the cerebral cortex [30, 31].

It is remarkable that, in contrast to the latency parameter, there were no changes for the amplitude in induced activity between target or standard stimuli, which suggests that the reduction of both alpha bands must be applied to standard or target stimuli without distinction of their relevance for the task. Moreover, the evoked activity was higher in amplitude than the induced activity (in absolute terms) for both types of stimulus and for both alpha bands. Klimesch et al [11] indicated that the alpha amplitude decrement will show higher or lower values considering the characteristics of the task (i.e., requiring more top-down or bottom-up processes). In our case, it appears that a top-down process (i.e., timing expectation, based on a fixed SOA) represents lower modulation than the evoked response caused by the presentation of the stimuli.

In any case, it is necessary to be cautious about amplitude results between the evoked and induced activity because of the possibility of mutual influence due to their phase values. In the present study, phase analyses allowed us to discard potential jittering effects between evoked and induced activity. However, this analysis has to be applied in every study due to possible random phase behaviors for both activities.

### Correlation analyses for map voltages

Evoked and induced activities for both alpha bands showed different signs that were present in the first 200 ms as has been described in other studies [21]. In a detailed study of correlation analyses, the r values were excellent for the comparison between first evoked and induced activity maps (<200 ms) in target and standard stimuli conditions and for both alpha bands. This result suggests that a very consistent modulation is found in the early evoked activity and is closely related to the induced modulation. It was not a goal of the present study to elucidate between diverse hypotheses for the psychophysiological implications of non-phase alpha activity (processing, inhibitory or inhibition-timing); however, the present results support previous studies in which phase and non-phase modulations coexist in the first 200 ms after the onset of the stimuli [21]. Moreover, considering the excellent correlation score between both activities, it seems reasonable to consider a dynamic balance in similar neural areas for visual processing (phase and non-phase). Evoked activity would represent the natural response caused by the income of the stimuli, and the decrement of induced activity would indicate the reduction of default alpha activity in closely related visual areas to improve the signal-to-noise ratio for visual processing. Nevertheless, the neurophysiological role of alpha modulations is still under debate, and the current experiments do not yield conclusive results. Our proposal is that the inhibition-timing hypothesis is in accordance with the present data. However, this interpretation cannot be taken as excluding other potential roles (processing or inhibitory). Future studies with different cognitive paradigms are needed to disentangle whether induced alpha modulations are always coupled (in time and topography) with the evoked responses.

In the evoked response, a second peak was observed only for the standard stimuli in both bands (low alpha: 660 ms; high alpha: 654 ms). A correlation analysis showed that the first evoked peaks for the target and standard were highly correlated with the second evoked peak for the standard (see Figs 3 and 4). Considering that lower amplitude is identified in the second peak and that the topography is closely related to the first evoked response, a plausible interpretation is that this second peak is a reevaluation of the standard stimuli after the response was performed. Another result that supports this interpretation originates from the high anticorrelation score found between the evoked and induced maps for standard stimuli (induced second valley: 698 and 728 ms for low and high alpha bands, respectively) (see Figs 3 and 4 for correlation values). It is possible that the reevaluation process implies a short term memory reactivation and forces to a new decrement of the alpha non-phase activity to facilitate the recall process. At this point it should be stated that the rebound of the evoked activity could be caused by the contribution of the offset components from own stimuli due to the fixed duration of the stimuli employed in the current study. The absence of a rebound in the target stimuli and in the standard stimuli in some subjects suggests that a more central (rather than sensorial) process is involved in the rebound. However, future studies would be needed to discard the offset contribution to the evoked response with a random stimulus duration experiment.

In the particular case of induced activity, a pattern in relevant stimuli previously described by other authors was identified for both alpha bands [32, 33]. This pattern consists of an early modulation (approximately 200 ms), a valley that extends from approximately 300 to 600 ms, and a later resynchronization that could be visible until 2000 ms, although in our case, the latter is limited to 900 ms because SOA was employed.

In the particular case of the induced activity associated with the standard stimuli, a different set of modulations was identified compared to the target stimuli. A first valley (low alpha: 144 and high alpha: 132 ms) showed low correlation scores with a positive peak observed later (low alpha: 518 ms, r: -0.344; high alpha: 522 ms, r: -0.652). However, the first valley correlated better with activity at longer latencies (low alpha: 698 ms, r: 0.821 and high alpha: 728 ms, r: 0.782), which suggests a similar neural configuration between the first and second valleys for standard stimuli. The increase in the correlation scores for the second valley, as previously discussed, could represent a reduction of alpha induced activity provoked by the reevaluation of standard stimuli (linked to the second positive peak for the standard evoked response).

In the correlation analysis inside the induced activity for target stimuli, a low correlation score was identified in low alpha (r: 0.473) for the comparison between the first and second valleys (120 and 448 ms). The explanation of this reduction of the correlation value is interpreted as a shift of the negativity to more parietal regions in the second valley compared to the first valley (more occipital). A considerable change in the topography suggests that different neural assemblies are involved in these activities and probably represent different functions. As the first valley seems to be more involved in sensory processing, the second valley could be more related (based on the longer latency and parietal topography) to the more central processes required for the target processing (i.e., attentional processes).

In the case of the high alpha band, a better correlation score was identified compared to the low alpha band for the comparison between the first and second valleys (124 and 440 ms) (r: 0.816). Therefore, it appears that both alpha bands could play different roles in the information processing, which has been proposed in previous studies [34]. In the current study, an induced high alpha band that exhibits a lower change in its correlation score between the first and second valleys could indicate that the decrement in the alpha amplitude represents a mechanism to improve the signal-to-noise ratio in sensory areas even in longer latencies (approximately between 400 and 500 ms). In the case of the induced low alpha band, a considerable change in the topography (indexed by a moderate correlation score) could represent a necessary decrement in the parietal areas related to the neural areas involved in more central cognitive processes. Previous studies have proposed that the low alpha band could be more related to attentional processes and the high alpha band may be linked to more sensory-semantic processing [34, 35, 36]. In any case, the differences between both bands were subtle and involved specific intervals. The complete set of alpha band results suggests that further studies are required to disentangle the potential role of both bands in information processing.

## Conclusions

The present study has evidenced multiple evoked and induced modulations in the cognitive processing of target and standard stimuli in a visual oddball task. The latency, amplitude (sign) and topography were diverse for each modulation, and specific functions were assigned to them. The first peak of evoked and first valley from induced (for both target and standards) were closely related in the latency and topography but with different amplitudes of magnitude and sign. The results in this case support those of previous studies in which a reduction of non-phase alpha activity could be involved in the increase of the signal-to-noise ratio for the processing of incoming stimuli (evoked response). In addition to this result, detailed analyses of subsequent modulations for evoked and induced activities in both types of stimuli (target and standard) have shown that there are more processes after these first modulations. The psychophysiological implications of these additional processes include a potential reevaluation of the standard stimuli manifested as a rebound in the late evoked response and a concomitant decrease in the induced activity. Moreover, a second valley in the induced activity for the target stimuli could represent a more central state of cognitive processing for relevant stimuli. Future studies are required to obtain a better understanding of all of these modulations and the neural sources that underlie them.
